# Distribution of Endophytic Bacteria in *Alopecurus aequalis* Sobol and *Oxalis corniculata* L. from Soils Contaminated by Polycyclic Aromatic Hydrocarbons

**DOI:** 10.1371/journal.pone.0083054

**Published:** 2013-12-17

**Authors:** Anping Peng, Juan Liu, Yanzheng Gao, Zeyou Chen

**Affiliations:** Institute of Organic Contaminant Control and Soil Remediation, College of Resource and Environmental Sciences, Nanjing Agricultural University, Nanjing, People's Republic of China; Institute for Plant Protection (IPP), CNR, Italy

## Abstract

The distributions of endophytic bacteria in *Alopecurus aequalis* Sobol and *Oxalis corniculata* L. grown in soils contaminated with different levels of polycyclic aromatic hydrocarbons (PAHs) were investigated with polymerase chain reaction followed by denaturing gradient gel electrophoresis technology (PCR-DGGE) and cultivation methods. Twelve types of PAHs, at concentrations varying from 0.16 to 180 mg·kg^−1^, were observed in the roots and shoots of the two plants. The total PAH concentrations in *Alopecurus aequalis* Sobol obtained from three different PAH-contaminated stations were 184, 197, and 304 mg·kg^−1^, and the total PAH concentrations in *Oxalis corniculata* L. were 251, 346, and 600 mg·kg^−1^, respectively. The PCR-DGGE results showed that the endophytic bacterial communities in the roots and shoots of the two plants were quite different, although most bacteria belonged to Firmicutes, Proteobacteria, Actinobacteria and Bacteroidetes. A total of 68 endophytic bacterial strains were isolated from different tissues of the two plants and classified into three phyla: Firmicutes, Proteobacteria and Bacteroidetes. In both plants, *Bacillus* spp. and *Pseudomonas* spp. were the dominant cultivable populations. With an increase in the PAH pollution level, the diversity and distribution of endophytic bacteria in the two plants changed correspondingly, and the number of cultivable endophytic bacterial strains decreased rapidly. Testing of the isolated endophytic bacteria for tolerance to each type of PAH showed that most isolates could grow well on Luria-Bertani media in the presence of different PAHs, and some isolates were able to grow rapidly on a mineral salt medium with a single PAH as the sole carbon and energy source, indicating that these strains may have the potential to degrade PAHs in plants. This research provides the first insight into the characteristics of endophytic bacterial populations under different PAH pollution levels and provides a species resource for the isolation of PAH-degrading endophytic bacteria.

## Introduction

Organic contaminants are frequently detected at relatively high concentrations in soils worldwide [1]. Some of these contaminants may be taken up by plants and translocated into shoots, which is the major pathway by which they reach the food chain [2], [3]. Polycyclic aromatic hydrocarbons (PAHs) are a class of environmental organic pollutants that are considered potentially extremely harmful owing to their toxic, mutagenic, and carcinogenic characteristics [4], [5]. A better understanding of how plants take up and metabolize PAHs from the soil could have considerable benefits for plant PAH risk assessments [6]. Therefore, methods for regulating and controlling the uptake and metabolism of PAHs in plants and effective measures for reducing plant PAH contamination risks have attracted much attention [7], [8].

Some chemicals, such as surfactants and ascorbic acid, can be used to regulate and control PAH absorption and metabolism processes in plants [9], [10]. However, most chemicals are not environmentally friendly and may cause secondary pollution. Additionally, the functionality of such chemicals is always limited by environmental conditions [11]. Previous studies have shown that many microorganisms associated with plants, such as arbuscular mycorrhizal fungi, biofilms on root surfaces, and endophytic bacteria, have enormous potential to degrade PAHs [12], and some of these microorganisms can even reduce PAH concentrations in plants [13]. Therefore, researchers have proposed an attractive strategy for reducing plant PAH contamination risks by utilizing plant-associated microecosystems to control the uptake and metabolism of PAHs by plants. This approach has been the focus of considerable interest in recent years [14].

Endophytic bacteria, defined as a class of microbes that reside within the interior tissues of plants without causing harm to host plants or environments [15], form one of the microbial communities most closely associated with plants. They have established harmonious associations with host plants during including symbiotic, mutualistic, commensalistic, and trophobiotic relationships over a long evolutionary process [16]. This assortment of bacteria have a wide variety of functions including the stimulation of plant growth [17], the promotion of biological nitrogen fixation [18], the protection of plants from harsh external environments, and the control of pathogen activities [19].

Previous studies have shown that some endophytic bacterial strains have the ability to degrade organic pollutants in plants and soils [20], [21]. Germaine et al. [22] inoculated pea plants with a 2,4-D-degrading endophytic bacterium (*Pseudomonas putida* VM1450) and found that this strain can internally colonize plants, maintain their growth, and cause a 24–35% increase in contaminant removal from the plants. Sheng et al. [14] isolated an endophytic pyrene-degrading bacterium *Enterobacter* sp. 12J1 from *Allium macrostemon* Bunge grown in PAH-contaminated soils and found that the bacterium increased plant resistance to pyrene by increasing plant biomass (from 13% to 56%) and promoted pyrene removal from pyrene-amended soils. Therefore, the use of endophytic bacteria to regulate the metabolism of organic pollutants and to reduce contamination risks in plants would be significantly advantageous [14].

Assessing the diversity, distribution, physiology, and ecology of endophytic bacteria in plants is prerequisite for isolating organic contaminant-degrading endophytic bacteria and using them to eliminate organic pollution in plants [23], [24]. There have been some reports regarding endophytic bacterial populations in plants grown in soils polluted with different contaminants [25], [26]. Moore et al. [23] investigated endophytic bacterial populations in poplar trees and found that a number of isolates had the ability to degrade BTEX compounds or to grow in the presence of TCE. Ho et al. [27] isolated 188 endophytic strains from three plants and found that among these strains, 29 not only grew well in the presence of naphthalene, catechol, and phenol but were also able to utilize the pollutant as a sole carbon source for growth. However, to our knowledge, little information is available about endophytic bacterial populations in plants from PAH-contaminated sites.

The aim of this study was to utilize PCR-DGGE technology combined with culture-dependent methods to investigate the distribution and diversity of endophytic bacteria in two plants (*Alopecurus aequalis* Sobol and *Oxalis corniculata* L.) that are common in China and also the dominant plants in a PAH-contaminated field. This is an indispensable precondition for the future isolation of functional endophytes to aid in the reduction of plant PAH pollution risk.

## Materials and Methods

### Chemical reagents

Sixteen PAH standards dissolved in acetonitrile were purchased from ShangHai Anpel Scientific Instruments (Shanghai, China) including naphthalene (NAP), acenaphthylene (ANY), acenaphthene (ANE), fluorine (FLU), phenanthrene (PHE), anthracene (ANT), fluoranthene (FLA), pyrene (PYR), benz[a]anthracene (BaA), chrysene (CHR), benzo[b]fluoranthene (BbF), benzo[k]fluoranthene (BkF), benzo[a]pyrene (BaP), indeno[1,2,3-cd] pyrene (InPy), dibenz[a,h]anthracene (DiahA), and benzo[ghi]perylene (BghiP). The concentration of each compound in the mixture was 200 mg·L^−1^.

The PAHs used in the tolerance test, including NAP, ACE, PHE, FLU, PYR, ANT, FLA, and BaP, were purchased from Aldrich Chemical Co. with purities >98%.

### Media

Beef extract peptone medium contained 5.0 g·L^−1^ beef extract, 5.0 g·L^−1^ NaCl, and 10.0 g·L^−1^ tryptone, pH 7.0. Luria-Bertani (LB) medium contained 10.0 g·L^−1^ tryptone, 5.0 g·L^−1^ yeast extract, and 10.0 g·L^−1^ NaCl, pH 7.0. Mineral salt medium (MSM) contained 1.50 g·L^−1^ (NH_4_)_2_SO_4_, 1.91 g·L^−1^ K_2_HPO_4_·3H_2_O, 0.50 g·L^−1^ KH_2_PO_4_, 0.20 g·L^−1^ MgSO_4_·7H_2_O,w=[to]?> and 1 mL of trace element solution (0.1 mg·L^−1^ CoCl_2_·6H_2_O, 0.425 mg·L^−1^ MnCl_2_·4H_2_O, 0.05 mg·L^−1^ ZnCl_2_, 0.01 mg·L^−1^ NiCl_2_·6H_2_O, 0.015 mg·L^−1^ CuSO_4_·5H_2_O, 0.01 mg·L^−1^ Na_2_MoO_4_·2H_2_O, 0.01 mg·L^−1^ Na_2_SeO_4_·2H_2_O). Plates containing solid media were prepared by adding 18 g·L^−1^ agar into the above-mentioned liquid media.

### Sample collection and PAHs analysis

Amur foxtail (*Alopecurus aequalis* Sobol), creeping oxalis (*Oxalis corniculata* L.), and soil samples were obtained from an aromatics factory in Nanjing (permission was obtained from the owner of this private land to perform the study on this site). Samples were collected from three stations (A, Z, and Q) at different distances from the aromatics factory. The physicochemical characteristics of the sampled soils were as follows: pH 5.87, 13.0% sand, 60.7% silt, 26.3% clay, and 1.36% organic matter. The total PAH contents of soils from stations A, Z, and Q were 178, 139, and 89.4 mg·kg^−1^, respectively. The plant samples were removed from the soil, carefully placed into a plastic bag, and immediately transported to the laboratory where the surface soil was scoured off [28].

Some of the soil and plant samples were freeze dried immediately for the determination of PAH contents. The PAHs were exacted from soil and plant samples as described previously by Ling and Gao [29]. The concentrations of PAHs were analyzed using high-performance liquid chromatography (HPLC) with a reverse-phase C_18_ column (Inertsil ODS-SP, 5 µm, 4.6×150 mm, GL Sciences Inc., Japan) using gradient elution. The recoveries of PAHs in the soil and plant samples that were investigated averaged between 85%–105% (n = 5, RSD ≤2.52%) after the entire procedure. Table 1 shows the concentrations of PAHs in soils.

**Table 1:** Concentrations of PAHs in soils.

PAHs	The concentrations of PAHs in soil (mg·kg^−1^ dry weight)		
	A	Z	Q
NAP	34.3±3.80a	25.5±7.30a	25.7±4.19a
ANE	42.8±1.77a	39.9±0.48ab	36.7±0.64b
PHE	8.62±0.67a	6.45±0.58a	3.36±0.78b
ANT	72.1±4.71a	56.6±4.34a	16.1±7.92b
FLU	0.89±0.02a	0.72±0.06b	0.73±1.44b
ANY	2.09±0.05a	1.01±0.04b	1.04±0.06b
PYR	1.68±0.02a	0.94±0.01b	0.91±0.03b
FLA	3.19±0.18a	2.14±0.09ab	0.82±0.34b
CHR	6.62±0.41a	3.20±0.04b	1.22±0.49c
BaA	0.94±0.01a	0.51±0.17b	0.52±0.01b
BbF	1.77±2.87a	0.86±4.41b	0.69±1.97b
BghiP	3.50±0.03a	1.64±0.04b	1.66±0.12b
∑PAHs	178±4.87a	139±4.20b	89.5±5.79c

Note: different letters in the same row indicate significant differences (P<0.05).

### Surface sterilization of plant samples

The plant samples were rinsed three times with deionized water and subsequently sterilized by sequential immersion in 75% (v/v) ethanol for 3–5 min, 2% sodium hypochlorite (v/v) for 3 min, and 70% ethanol for 30 sec. Finally, the plant samples were washed three times with sterilized distilled water to remove surface sterilization agents [28]. To determine whether the surface disinfection process was successful, plants were pressed onto fresh beef extract peptone agar plates to detect any remaining epiphytic bacteria.

### PCR-DGGE analysis

Total DNA extraction was performed according to the protocol described by Hung et al. [30]. The 16S rDNA V3 sequences were amplified by PCR using the extracted genomic DNA as a template and the bacterial universal primers 341f (with a GC clamp; 5′- CGCCCGCCGCGCGCGGCGGGGGGCGGGGGCACGGGGGGCCTACGGGAGGCAGCAG-3′) and 534r (5′- ATTACCGCGGCTGCTGG-3′). The PCR mixture (25.0 µL) contained 1 µL of DNA template (5 ng µL^−1^), 12.5 µL of Premix Taq (TaKaRa, Premix Taq Version 2.0), 0.5 µL of primers (12.5 µg·μL^−1^), and 1 µL of bovine serum albumin (20 µg·μL^−1^). The PCR was performed in a DNA Engine Thermal Cycler (TaKaRa, D-8308), and the PCR program consisted of an initial denaturation at 94°C for 5 min, 30 cycles at 94°C for 30 sec, 60°C for 30 sec, and 72°C for 30 sec, followed by a final extension step at 72°C for 10 min.

DGGE analysis was performed with a Dcode Multiple System (Bio-Rad Laboratories Inc., Hercules, CA, USA) using the following protocol: Aliquots (25 µL) of the PCR products were loaded onto an 8% (w/v) polyacrylamide gel with a denaturant gradient ranging from 40% to 65%. Electrophoresis was run for 16 h at 120 V and 60°C in 1× TAE buffer (40 mM Tris, 20 mM acetic acid, and 2 mM EDTA), after which the gels were soaked for 30 min in SYBR Green I nucleic acid stain (1∶10,000 dilution) and immediately photographed under UV light. Specific bands were excised from the DGGE gel and washed twice with sterilized distilled water. Each band was used as a direct template for PCR to recover the DNA fragment separated by DGGE. The PCR conditions were the same as those used for the original PCR. The fragments recovered from the PCR were subjected to DGGE again to confirm the equality of their mobility. If a single band appeared in a DGGE gel for one sample, the PCR products were purified with the PCR Cleanup Kit (Axygen, USA) and used for direct sequencing (Invitrogen). When multiple bands appeared in one sample, the bands were repeatedly electrophoresed and excised until only a single band was detectable on the DGGE gel.

### Isolation of cultivable endophytic bacteria

After surface disinfection, the root and shoot tissues of plants were cut into pieces and triturated in 5 mL of sterile distilled water. Subsequently, 100 µL aliquots of the appropriate dilutions (10^−1^, 10^−2^, and 10^−3^) were spread onto beef extract peptone medium and incubated at 30°C for 7 days.

Each bacterial strain with a different colonial morphology was identified by 16S rDNA sequence analysis. Genomic DNA was extracted from each isolated endophytic bacterium with a DNA Extraction Kit (Axygen, USA). The PCR mixture was the same as that used for 16S rDNA V3 sequence amplification except that the primers were 27f (5′-AGAGTTTGATCCTGGCTCAG-3′) and 1492r (5′-GGTTACCTTGTTACGACT T-3′) [31], and the annealing temperature was altered to 52°C.

### DNA Sequence Analysis and Accession Numbers

Analysis of 16S rDNA sequences was performed using the NCBI database and nucleotide BLAST (http://blast.ncbi.nlm.nih.gov/Blast.cgi). The sequences were aligned using the ClustalW program. Sequences were identified as the most closely related species with the highest similarity. A phylogenetic tree was constructed using evolutionary distances based on the 16S rDNA V3 sequences with the neighbor-joining method [32]. Tree topologies were evaluated by performing bootstrap analysis of 1,000 datasets with the MEGA 5.05 package.

The 77 fragments of 16S rDNA V3 sequences determined in this study were deposited in the GenBank database under the accession numbers KF051455–KF051531, and the 68 pieces of 16S rDNA sequences determined in this study were deposited with the accession numbers JX994089–JX994132 and JX994134–JX994157.

### Tolerance of isolated endophytic bacteria to each type of PAH

A suspension of each isolated endophytic bacterial strain was plated on MSM or LB agar plates containing one of the following PAHs: NAP (with a final concentration of 100 mg·L^−1^), PHE, FLU, ANT, ACE, PYR, FLU (with final concentration of 30 mg·L^−1^), or BaP (with a final concentration of 10 mg·L^−1^), the concrete method was referring to Yao et al. [33]. The plates were incubated for 3–7 days at 30°C, and bacterial growth was monitored regularly to investigate the tolerance of each endophytic bacterial strain to each type of PAH.

### Statistical analyses

All data collected were processed using Microsoft Excel 2007. Each data point is the mean of at least three replicates, and error bars represent standard deviations (SD). The data were statistically analyzed using analysis of variance (ANOVA) with the statistical package SPSS 13.0. Differences were considered significant at p values <0.05, and standard deviations obtained from three parallel samples are shown in the figures as error bars.

## Results

### PAH concentrations in plants from PAH-contaminated soils

In this study, 12 types of PAHs that have been designated by the US Environmental Protection Agency as priority pollutants were detected in soil and plant samples, including NAP, ANE, PHE, ANT, FLU, ANY, PYR, FLA, CHR, BaA, BbF, and BghiP. As shown in Table 1, soil samples obtained from each sampling station contained different concentrations of PAHs, and the total PAH contents of soils from A, Z, and Q stations were 178, 139, and 89.5 mg·kg^−1^, respectively. ANOVA revealed significant differences among the total PAH concentrations at the three sampling stations. ANE and ANT were the main components in all three soil samples, accounting for 23.9–40.9% and 18.0–40.6% of the total concentrations, respectively.

The 12 types of PAHs detected in rhizosphere soils were also determined in the roots and shoots of the two plants (Tables 2 and 3), and the overwhelming majority of PAHs accumulated in plant roots. For example, at position A, PAHs were enriched to a concentration of 217 mg·kg^−1^ in the roots of *A. aequalis*; however, in the shoots, the total PAH concentration was only 88.0 mg·kg^−1^. Furthermore, for both plants, as soil PAH concentrations increased, the PAH concentrations in the plants also increased. As shown in Tables 2 and 3, the PAH concentrations in the roots of plants from station A were significantly higher than those in plants from the other two stations. Two- to three-ring PAHs were the main detectable pollutants in plant samples, with proportions of 94.9–96.2% in *A. aequalis* and 87.8–94.2% in *O. corniculata* grown in differentially contaminated soils. Conversely, four- to six-ring PAHs accounted for only minor proportions of the total PAHs in the two plants (approximately 3.8–5.1% and 5.8–12.2%, respectively).

**Table 2:** Concentrations of PAHs in *Alopecurus aequalis*.

PAHs	Root (mg·kg^−1^ dry weight of plant)			Shoot (mg·kg^−1^ dry weight of plant)		
	A	Z	Q	A	Z	Q
NAP	120±5.43a	45.5±8.11b	59.9±21.05ab	42.8±5.30a	26.9±5.34a	20.2±7.82a
ANE	33.9±7.81a	28.8±6.26a	24.9±7.80a	15.9±3.36a	12.5±0.83a	9.32±1.85a
PHE	4.85±0.21a	4.12±0.01a	3.7±1.31a	2.31±0.28a	2.25±0.03a	1.98±0.71a
ANT	49.0±2.73a	44.3±6.64a	30.5±9.02a	22.6±3.80a	20.7±1.39a	23.2±3.93a
FLU	0.44±0.01a	0.39±0.01a	0.36±0.13a	0.21±0.12a	0.22±0.01a	0.19±0.06a
ANY	0.73±0.04a	0.63±0.05a	0.56±0.22a	0.39±0.10a	0.45±0.10a	0.34±0.07a
PYR	0.62±0.04a	0.59±0.07a	0.48±0.06a	0.30±0.02a	0.27±0.01a	0.30±0.01a
FLA	1.72±0.04a	1.54±0.22a	1.28±0.24a	0.82±0.12a	0.75±0.07a	0.81±0.04a
CHR	3.78±0.37a	2.90±0.15ab	1.81±0.57b	1.77±0.05a	1.66±0.34a	1.61±0.08a
BaA	0.32±0.01a	0.29±0.03a	0.25±0.02a	0.16±0.01a	0.17±0.00a	0.16±0.00a
BbF	0.54±0.10a	0.51±0.04a	0.43±0.13a	0.31±0.03a	0.34±0.02a	0.31±0.04a
BghiP	0.77±0.01a	0.71±0.04a	0.60±0.10a	0.38±0.01a	0.36±0.00a	0.38±0.01a
∑PAHs	217±11.7a	130±12.0b	124±2.30b	88.0±3.97a	66.5±2.41b	58.8±4.08c
CF_PAHs_	1.21	0.93	1.39	0.49	0.48	0.66

Note: different letters in the same row indicate significant differences (P<0.05); CF_PAHs_ means the plant concentration factors of total PAHs.

**Table 3:** Concentrations of PAHs in *Oxalis corniculata*.

PAHs	Root (mg·kg^−1^ dry weight of plant)			Shoot (mg·kg^−1^ dry weight of plant)		
	A	Z	Q	A	Z	Q
NAP	109±6.53a	64.1±12.01b	63.3±19.57b	49.2±2.78a	45.8±15.76a	19.7±10.91b
ANE	83.2±3.09a	40.2±2.14b	38.5±3.56b	30.2±3.71a	32.3±10.61a	12.9±3.71b
PHE	18.2±2.83a	9.28±0.34b	8.4±2.47b	5.25±0.32a	4.53±1.53ab	2.26±0.54b
ANT	180.±10.12a	85.8±0.42b	68.5±9.88c	47.2±1.41a	41.3±3.13a	19.6±4.13b
FLU	1.69±0.06a	0.78±0.02b	0.81±0.21b	0.48±0.04a	0.48±0.19a	0.29±0.02a
ANY	2.22±0.40a	1.16±0.09b	1.07±0.30b	0.71±0.08a	0.75±0.22a	0.40±0.01a
PYR	2.32±0.88a	1.12±0.07b	1.14±0.27b	0.68±0.02a	0.61±0.20a	0.36±0.02b
FLA	6.01±1.12 a	2.97±0.28b	1.67±1.26c	1.75±0.08a	1.44±0.63ab	0.64±0.27b
CHR	50.0±6.58a	6.18±3.83b	5.83±1.22b	3.58±0.30a	2.78±1.36a	1.45±0.96a
BaA	1.22±0.24a	0.60±0.09b	0.60±0.17b	0.37±0.02a	0.31±0.09a	0.21±0.01a
BbF	2.28±0.49a	1.13±0.15b	0.60±0.25c	0.69±0.06a	0.50±0.04a	0.44±0.06a
BghiP	3.21±0.67a	1.58±0.08b	1.48±0.35b	0.89±0.03a	0.77±0.09ab	0.45±0.03b
∑PAHs	459±10.6a	215±14.2b	192±23.0b	141±5.74a	132±12.4a	58.7±9.34b
CF_PAHs_	2.57	1.54	2.15	0.79	0.94	0.66

Note: different letters in the same row indicates a significant differences (P<0.05); CF_PAHs_ means the plant concentration factors of total PAHs.

However, there were obvious differences in the absorption and accumulation of PAHs between the two plants. *O. corniculata* was better able to accumulate PAHs compared with *A. aequalis*. For example, the PAH concentrations in *O. corniculata* grown at positions A, Z, and Q were 251, 346, and 600 mg·kg^−1^, respectively, which were significantly higher than those in *A. aequalis* (184, 197, and 304 mg·kg^−1^, respectively at positions A, Z, and Q). Additionally, the concentration of NAP in *A. aequalis* was the highest among all detected PAHs, accounting for 36.8–53.4% of the total PAH content of the plants from the different stations. In contrast, the concentration of ANT was highest in *O. corniculata*, accounting for 60.9–62.5% of the total PAH content.

### DGGE analysis of the endophytic bacterial community

**Figure 1:**
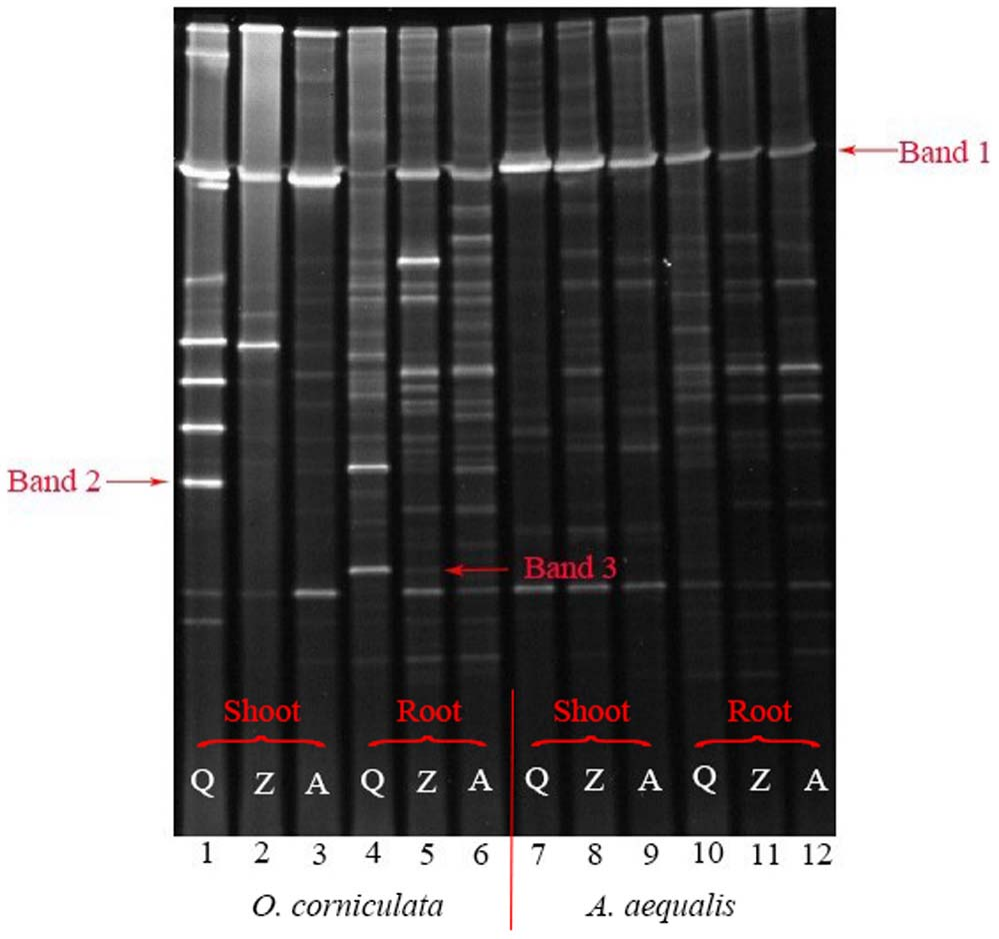
Representative DGGE for PCR-amplified 16S rDNA V3 fragments from endophytic bacteria in *Alopecurus aequalis* and *Oxalis corniculata*. The genus of the band marked in the figure: Band 1 - *Pseudomonas* sp; Band 2 - uncultured bacterium clone; Band 3 - *Nesterenkonia* sp.

**Figure 2:**
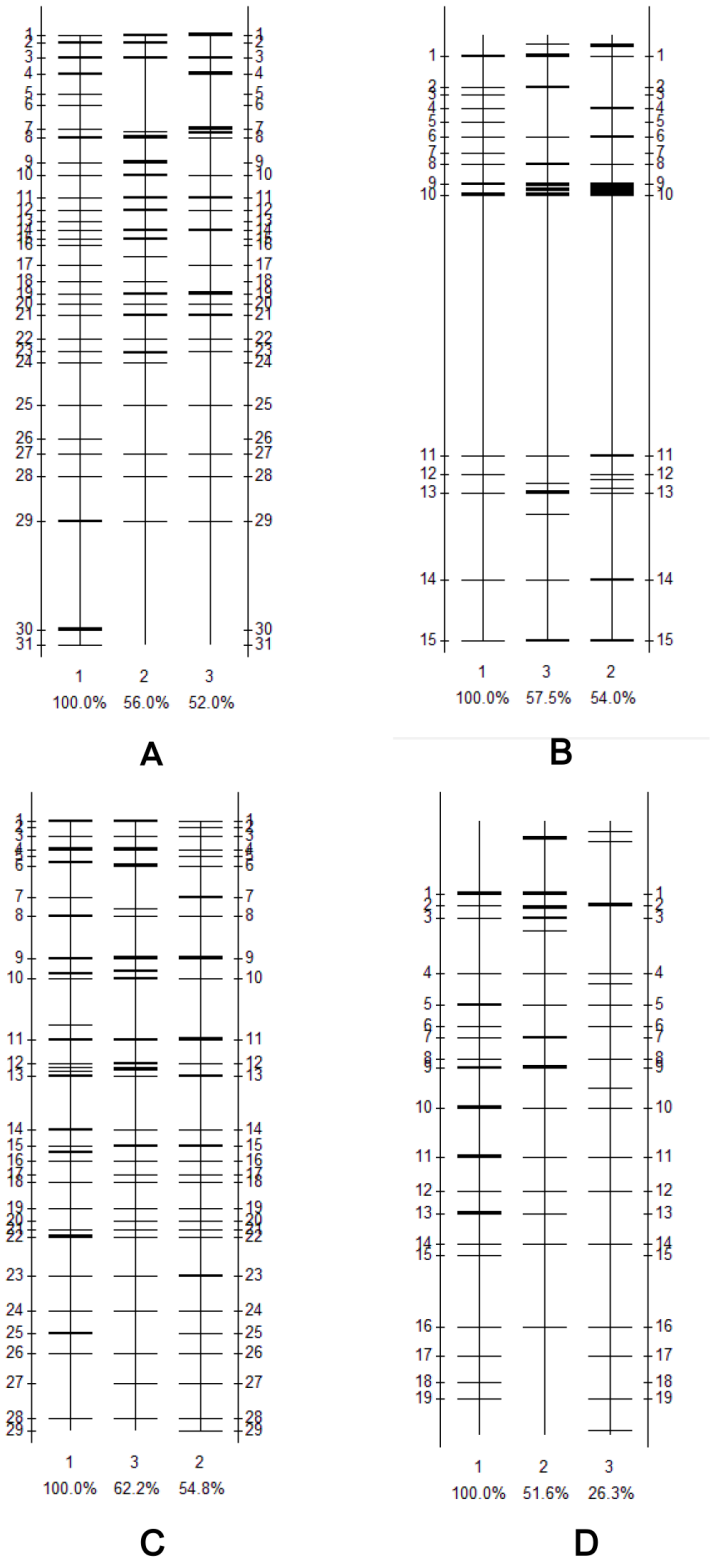
The similarity of endophytic bacterial community in roots of *Alopecurus aequalis* (A), shoots of *Alopecurus aequalis* (B), roots of *Oxalis corniculata*(C) and shoots of *Oxalis corniculata* (D). 1- Q pollution level (defined as 100%), 2- Z pollution level, 3-A pollution level.

The PCR-DGGE profiles are shown in Figure 1. All plant samples from the three sites contained band 1 (*Pseudomonas* sp.), indicating that one of the dominant bacteria was the same in both plants at different levels of PAH contamination. However, there were also some discrepancies between different plant tissues and different places. For example, band 2 (uncultured bacterium clone) was only existed at site Q, and band 3 (*Nesterenkonia* sp.) was only existed at sites Q and Z. Pollution intensity could significantly influence the diversity of the endophytic bacterial community. As shown in Figure 2, the plants that grew in lightly polluted soils consistently showed the highest diversity.

Nearly all the bands found in the DGGE gel were sequenced, and after removing the 16S rDNA and 18S rDNA of mitochondria and chloroplasts, we managed to obtain exactly 77 different bacterial sequences (Tables S1 and S2 in File S1), among which 56 were isolated from *A. aequalis* (root, 37; shoot, 22; both, 3) and 32 were obtained from *O. corniculata* (root, 27; shoot, 12; both, 4). Overall, 44.1% of the sequences isolated from *A. aequalis* were derived from uncultured bacteria, 8.47% were derived from *Pseudomonas* sp. and 6.78% were derived from *Halomonas* sp. The remaining 40.7% were derived from approximately 20 different genera of bacteria. In *O. corniculata*, the highest percentage of sequences (30.6%) was also derived from uncultured bacteria, followed by *Pseudomonas* sp. (19.4%) and *Enterobacter* sp. (8.33%). The remaining 41.7% of sequences were derived from 15 different genera of bacteria.

**Figure 3:**
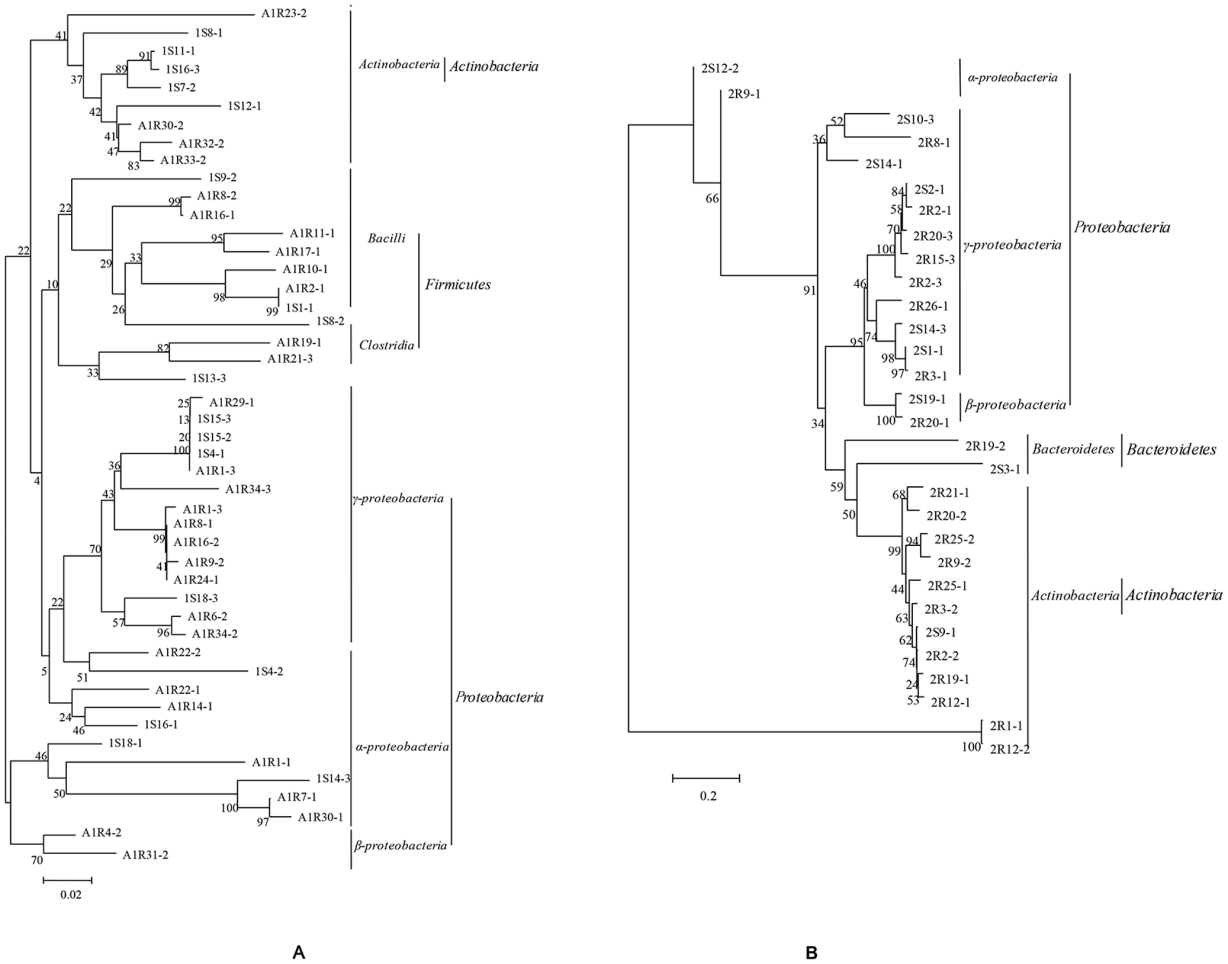
Phylogenetic trees by 16S rDNA V3 sequence analysis of endophytic bacterial community of *Alopecurus aequalis* (A) and *Oxalis corniculata* (B). The codes with characters and numbers indicate the isolated DGGE bands. Bootstrap values are shown for each node in a bootstrap analysis of 1,000 replicates.

Phylogenetic analysis (Figure 3) of the endophytic bacterial community performed using the results of the DGGE gel indicated that these bacteria belong to four phyla (Firmicutes, Proteobacteria, Actinobacteria and Bacteroidetes), seven classes (*Bacilli, Clostridia, α-proteobacteria, β-proteobacteria, γ-proteobacteria, Actinobacteria, Bacteroidetes*), and over 30 families. Most of the isolates from *A. aequalis* belonged to Proteobacteria and Firmicutes, and the remaining belonged to Actinobacteria. Proteobacteria also composed a large percentage of the bacteria isolated from *O. corniculata*, and the remaining bacteria from *O. corniculata* belonged to Bacteroidetes and Actinobacteria.

### Cultivable endophytic bacterial populations in plants from PAH-contaminated soils

A total of 68 species of endophytic bacteria were isolated and identified, among which 39 were isolated from *A. aequalis* (root, 27; shoot, 20; both, 7), 28 were isolated from *O. corniculata* (root, 15; shoot, 17; both, 3) and 1 was isolated from both plants. The isolates were identified using 16S rDNA analysis, and these 16S rDNA sequences shared high identities with their most closely related species in the database (≥97%), with most having identities of 99–100% with known bacterial species (Tables S3 and S4 in File S1).

The cultivable endophytic bacterial populations of *A. aequalis* and *O. corniculata* grown in PAH-contaminated soils showed low diversity. The 68 endophytic bacterial species were classified into 14 genera belonging to five classes, including *Bacilli*, *α-proteobacteria*, *β-proteobacteria*, *γ-proteobacteria*, and *Flavobacteriaceae*. Although more endophytic bacterial species were isolated from *A. aequalis* than from *O. corniculata*, the endophytic bacterial population in *O. corniculata* included more genera than that in *A. aequalis*. In *A. aequalis*, *Bacillus* and *Pseudomonas* spp. constituted a large proportion, accounting for 65.5% and 24.1%, respectively, of the total bacterial population. Meanwhile, members of *Bacillus* spp. accounted for the highest proportion of strains in *O. corniculata*, constituting 43.9% of the total bacterial population, followed by *Pseudomonas* spp. and *Rahnella* spp., each constituting 21.1% of the total population.

### Amounts of cultivable endophytic bacteria in plants from PAH-contaminated soils

**Figure 4:**
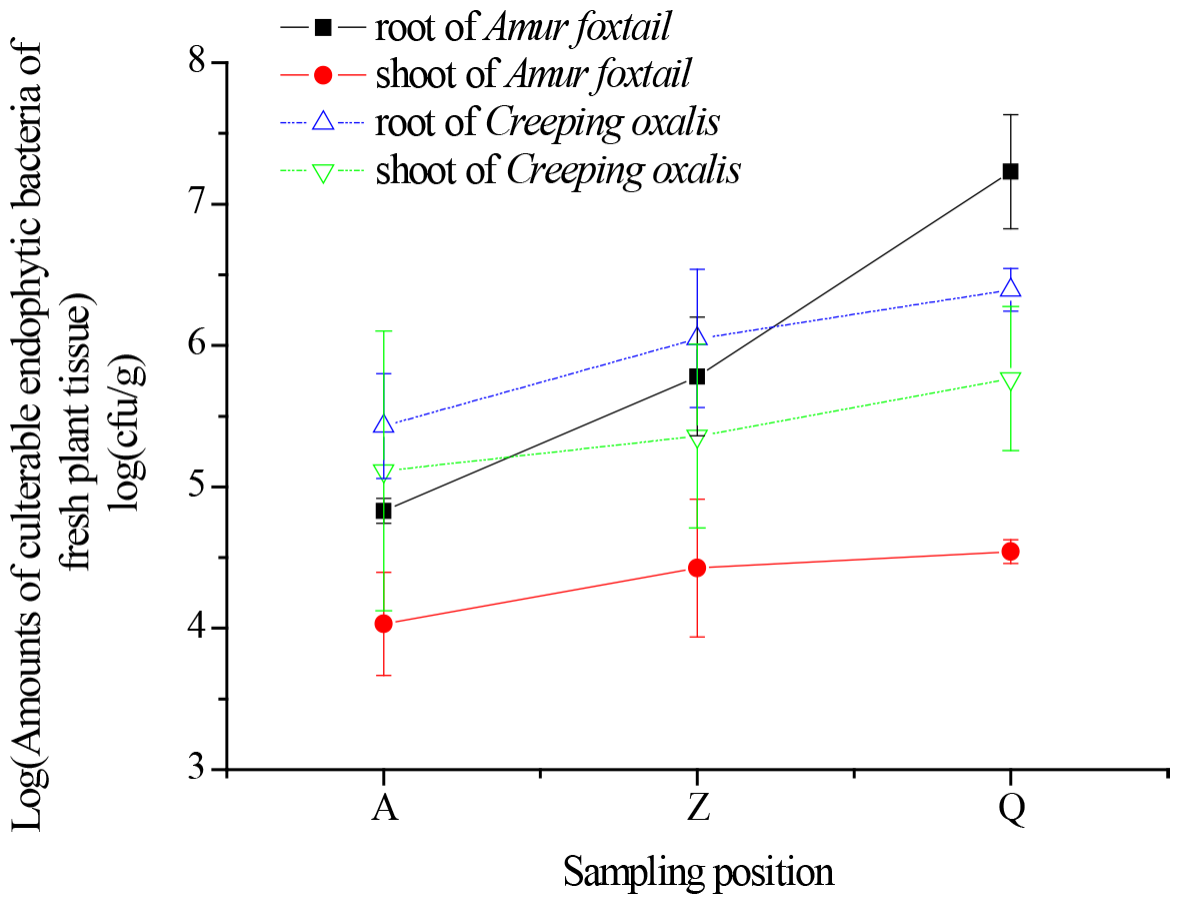
Amounts of cultivable endophytic bacterial strains in *Alopecurus aequalis* and *Oxalis corniculata* under different PAH pollution levels.

As shown in Figure 4, the cultivable endophytic bacteria detected in the two studied plants were on the order of 10^4^ to 10^7^ CFU per gram of fresh plant tissue in most cases, and the number of endophytic bacteria in roots was higher than that in shoots. As the PAH concentration increased, the total number of cultivable endophytic bacterial strains was reduced. For example, the number of endophytic bacterial strains in *A. aequalis* from station Q was 251 and 27 times larger than those from stations A and Z, respectively, but the concentration of PAHs was only 0.5 and 0.8 times of those from stations A and Z, respectively. Furthermore, as PAH concentrations increased, the total number of endophytic bacterial cells in plant tissues decreased by different degrees, and the range varied more in plant roots compared with shoots. For example, the number of endophytic bacteria in *A. aequalis* roots from station Q was 351 times that of roots from station A, while in shoots, the difference was only 3.23 times greater at station Q compared with station A.

**Figure 5:**
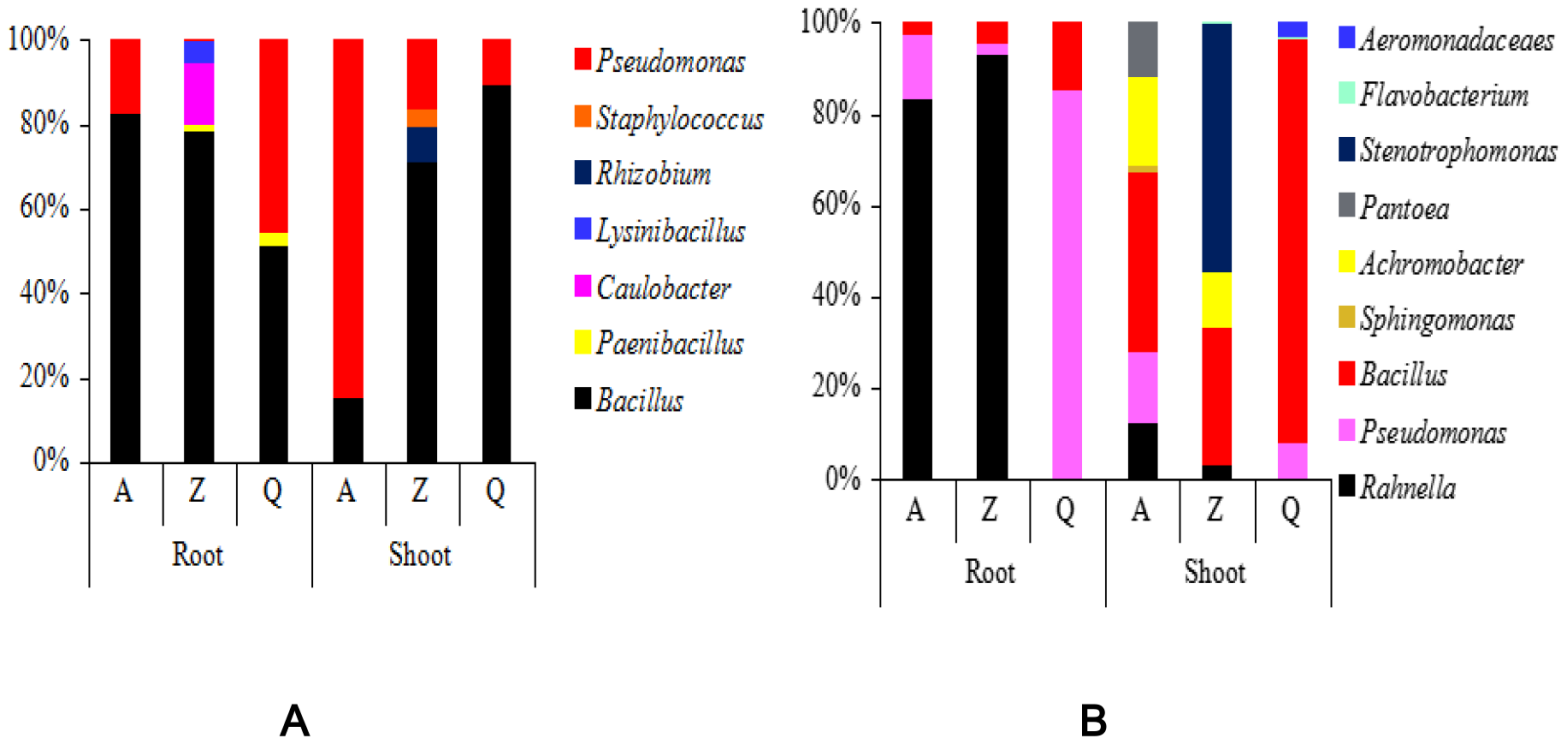
Amounts of cultivable endophytic bacterial strains belonging to each genus in *Alopecurus aequalis* (A) and *Oxalis corniculata* (B) under different PAH pollution levels.

PAH pollution levels also had impacts on the dominant populations of cultivable endophytic bacteria in the two plants (Figure 5). As PAH concentrations increased, the proportion of *Bacillus* spp. and *Pseudomonas* spp. showed opposite tendencies in the roots and shoots of *A. aequalis*. For example, under higher PAH concentrations (station A), *Bacillus* spp. accounted for 82.4% of the total endophytic bacteria in roots, and at the other two stations, *Bacillus* spp. accounted for 78.5% (Z) and 51.4% (Q). However, *Bacillus* spp. in the shoots of *A. aequalis* showed the opposite trend, with proportions of 15.4%, 70.8%, and 89.4% in samples from stations A, Z, and Q, respectively. Meanwhile, with increasing pollution levels, the proportion of *Pseudomonas* spp. decreased in plant roots and increased in shoots. The dominant populations in *O. corniculata* from different stations were quite different. The dominant genus in roots was *Rahnella* spp. when the pollution level was relatively high (A, Z); however, under a lower level of pollution, the dominant genus became *Pseudomona*s spp.

### Tolerance of the isolated endophytic bacteria to each type of PAH

Most of the isolates from the two plants were able to grow well on LB medium containing different PAHs, indicating that they were very tolerant to different PAHs. Some of the isolates were even able to grow rapidly on MSM medium with PAHs as the sole carbon and energy source, illustrating their potential for the degradation of various PAHs (Table S5–S8 in File S1).

Nine bacterial isolates obtained from the roots of *A. aequalis* grew well on MSM medium containing NAP. Eleven, seven, eight, and ten strains grew normally on MSM medium in the presence of tricyclic ANE, FLU, PHE and ANT, respectively, and ten and five strains grew well on MSM medium containing tetracyclic PYR and FLA, respectively. Only three bacterial strains grew normally on MSM medium with 10 ppm BaP (AF10, AF13, and AF25), and AF13 was the only strain that grew normally on MSM medium with each tested PAH. Many of the isolates from *A. aequalis* stems grew well on MSM medium with PAHs, and four strains even showed high tolerance to six or seven types of PAHs; however, none of the strains were tolerant to all tested PAHs.

Among all the strains isolated from *O. corniculata* roots, only CO3 grew well on MSM medium with five types of PAHs. Five isolates grew normally on MSM medium containing NAP, and two, six, two, and three strains grew on MSM medium in the presence of tricyclic ANE, FLU, PHE, and ANT, respectively. One and two strains grew well on MSM medium containing tetracyclic PYR and FLA, respectively. Only one bacterial strain grew normally on MSM medium with 10 mg·L^−1^ BaP. Some of the isolates from *O. corniculata* shoots grew well on three or four types of PAHs, but only CO26 grew normally on MSM medium with more than five types of PAHs.

## Discussion

Previous studies have shown that many plants have the ability to uptake and accumulate organic pollutants [2], [3], [7]. Gao and Zhu [34] investigated the capacity of 12 plant species to absorb and accumulate PHE and PYR and found that the RCFs (defined as the ratio of the PAH concentration in roots to that in soils on a dry weight basis [35]) of PHE and PYR for plants grown in contaminated soils were 0.05–0.67 and 0.23–4.44, respectively, whereas the SCFs (defined as the ratio of the PAH concentration in shoots to that in soil on a dry weight basis) of PHE and PYR were 0.006–0.12 and 0.004–0.12, respectively. In this study, we also found that both *A. aequalis* and *O. corniculata* showed strong abilities to absorb and enrich various types of PAHs (Tables 2 and 3). The RCFs of total PAHs for *A. aequalis* and *O. corniculata* were 0.93–1.39 and 1.54–2.57, respectively, and the SCFs of the two plants were 0.48–0.66 and 0.66–0.94, respectively.

Interestingly, the total PAH concentration in *O. corniculata* was significantly higher than that in *A. aequalis*. This may be attributable to the different growing seasons (*O. corniculata* is a perennial herb whereas *A. aequalis* is an annual herb) or the different lipid contents of the two plants [36], [37]. Chiou et al. [38] built a partition-limited model indicating that water-insoluble contaminants, even in small amounts, were the major compounds present in the plant-lipid phase. Zhu and Gao [39] also confirmed a significant positive correlation between the root concentration of phenanthrene and root lipid content. Interestingly, both *A. aequalis* and *O. corniculata* absorbed few high molecular weight PAHs with larger numbers of benzene rings (pentacyclic and hexacyclic), which could be due to the poor bioavailability of these types of PAHs [40].

It is well known to use the surface sterilization methods to remove the epiphytic bacteria before the isolation of endophytic bacteria [28], [41], [42]. In this study, to determine whether the surface disinfection process was successful, plants and water from the final rinse were both pressed onto fresh beef extract peptone agar plates to detect any remaining epiphytic bacteria. No epiphytic bacteria were detected, indicating that the surface disinfection was successful, and the 68 isolated bacterial strains associated to the plants in this study were all endophytic bacteria. The several washes that have been done after sterilization should also ensure the absence of epiphytic dead bacterial cells, which could be detected by PCR-DGGE method. However, additional experiments such as fluorescence *in situ* hybridization (FISH) or scanning electronic microscopy (SEM) observations could be needed to exclude the presence of epiphytic bacteria.

Although endophytic bacteria have been previously reported in many plants including sugarcane [43], ginseng [44], and aquatic plants [45], among others, little information is available regarding endophytic bacteria in plants grown in PAH-contaminated soils. In this study, a total of 68 endophytic bacterial strains were isolated in *A. aequalis* and *O. corniculata* growing in PAH-contaminated stations. As known to us, Bacilli are a group of the commonest bacteria associated with plants and take a certain proportion of the cultivable endophytic bacteria in lots of plants [45]–[47]. In our study, members of *Bacillus* spp. account for the highest proportion of cultivable endophytic bacterial strains in both *Alopecurus aequalis* Sobol and *Oxalis corniculata* L, followed by *Pseudomonas* spp.. Many bacterial strains belonging to these two genera are known to be capable of degrading various types of organic pollutants, which accounts for the frequent occurrence of these strains in organically contaminated soils in relatively high amounts [23]. In addition, the numbers of endophytic bacteria in the two plants were quite high: 7.84×10^4^−1.70×10^7^ and 4.40×10^5^−3.06×10^6^ cfu·g^−1^ in *A. aequalis* and *O. corniculata*, respectively. In contrast, Garbeva et al. [48] and Araújo et al. [49] obtained only 1.0×10^3^−1.0×10^5^ cfu·g^−1^ and 10^3^−10^4^ cfu·g^−1^ of endophytic bacterial cells from the potato and citrus plants, respectively. Some endophytic bacterial strains, such as strains AF14, AF19, and CO4, occurred in both the roots and shoots of a given plant, indicating that endophytic bacterial strains can be transported and spread throughout plant systems[50], [51].

Compared with culture-dependent method, using PCR-DGGE technology we found that bacterial community profiles showed extensive variability depending on the plant origin, tissue, and PAH levels in soil. To further explore this dissimilarity, the Shannon diversity index was calculated for each sample using Quantity One and Bio-Dap software. The results show that from Q to A, the Shannon diversity indices of *A. Aequalis* were 3.38, 3.2, and 2.93 in roots and 2.55, 3.03, and 2.74 in shoots, respectively. In *O. Corniculata*, the Shannon diversity indices were 3.34, 3.27, and 3.29 in roots and 2.78, 2.66, and 2.73 in shoots. The Shannon index includes two basic components: abundance and evenness of the species present [52], and this result indicated that different plant samples and pollution levels could lead to different bacterial distributions and levels of diversity. Qing et al. [53] and Robson et al. [54] also obtained a similar conclusion in their studies. Through phylogenetic analysis (Figure 3), we can see that the most prominent groups in the two plants were both related to *Proteobacteria*, which agrees with other studies [55], [56].

Plant-associated habitats are dynamic environments in which many factors affect the species compositions of microbial communities [57]. The host plant species is one of the major influencing factors [58]. For instance, Chen et al. [45] studied endophytic bacterial species of four aquatic plants, and their results showed that the dominant endophytic bacterial taxa in the four plants were quite different; *Pseudomonas* spp. and *Staphylococcus* spp. were the dominant taxa in *Phragmites communis* and *Potamogeton crispus*, whereas in *Nymphaea tetragona* and *Najas marina*, the dominant taxa were *Aeromonas* spp. and *Bacillus* spp. In this study, at the same pollution level, the dominant endophytic bacteria in *A. aequalis* and *O. corniculata* were also different. Pollution stress within a plant growing area is another major environmental factor influencing endophytic bacterial communities [59]. Sobral et al. [28] found that many endophytic bacteria could be cultivated from soybeans grown in soil to which glyphosate had been previously applied (pre-planting); however, when the glyphosate was enriched, the taxa of the cultivable endophytic bacteria changed. Similar results were observed in this study: The proportion of each endophytic bacterial genus in a given plant was found to vary at different pollution levels. Even among different tissues of one plant, when the total PAH concentration varied, the endophytic bacterial population changed correspondingly (Figure 5).

Studying endophytic bacterial populations under different pollution levels is a popular means of examining contaminant-degrading bacterial flora [21], [23]. Siciliano et al. [60] found that the amount of contaminant-degrading bacterial cells increased with exposure to a contaminated environment. Phillips et al. [59] analyzed the relationship between the endophytic bacterial community and the capacity for organic pollutant degradation in alfalfa. These authors found that when the dominant population consisted of *Pseudomonas* spp., the ability of plants to metabolize alkane pollutants improved, whereas when the dominant bacterial populations shifted to *Brevundimonas* spp. and *Pseudomonas rhodesiae*, the capacity of the plant to metabolize aromatic hydrocarbons increased. This suggests that the dominant endophytic bacterial taxa isolated from contaminated plants may have the potential to degrade PAHs in plants [23], [45]. Thus, further studies of the tolerance of isolated endophytic bacterial strains to various PAHs were performed.

Many endophytic bacterial strains have been reported to be tolerant of PAHs, and some of these can even degrade various PAHs by using them as sole carbon and energy sources for cell growth [14], [22], [40]. Furthermore, previous studies have proposed that endophytic bacteria could assist plants in remediating organic pollutants, mainly through direct metabolism of the organic pollutants [61], the promotion of plant growth and reduction of plant disease [40], the regulation of plant enzyme system activity and the promotion of metabolic gene expression [62]. Accordingly, researchers have proposed that identifying endophytic bacteria that degrade PAHs and inoculating them into host plants grown at PAH-contaminated sites would be of great significance for reducing plant PAH pollution risks [63]. In this study, some isolates showing strong tolerance to many types of PAHs were identified. However, much work remains to be done before these bacteria can be used for plant PAH pollution reduction. First, the PAH-degrading abilities of each screened endophyte must be confirmed, and the functional strains should then be inoculated into target plants. Lastly, the impacts and mechanisms of PAH-degrading endophytes on the uptake of PAHs by plants must be clarified.

## Conclusions

*Alopecurus aequalis* and *Oxalis corniculata* grown in PAH-contaminated sites were selected to investigate the concentrations of various PAHs and the distributions of endophytic bacteria in plant roots and shoots. The results revealed that both plants could absorb and accumulate PAHs in the roots and shoots, with *O. corniculata* exhibiting the higher enrichment ability. The endophytic bacterial communities in the roots and shoots of the two plants were quite different, although most of these bacteria belonged to Firmicutes and Proteobacteria. As pollution increased, the diversity and distribution of endophytic bacterial strains in both plants changed correspondingly, while Proteobacteria always accounted for a large percentage of the total, and the number of cultivable endophytic bacterial strains decreased rapidly. Most bacterial isolates from the two plants showed strong tolerance to different PAHs, and some of them were able to grow rapidly with PAHs as their sole carbon and energy sources, indicating that these strains may have the potential to degrade PAHs in plants. It's represented a significant opportunity to study how to reduce plant PAH contamination risk and remediate PAH-contaminated soils.

## Supporting Information

File S1 **Contains Tables S1–S8. Table S1.** Identified 16S rDNA V3 sequences in *Alopecurus aequalis*. **Table S2.** Identified 16S rDNA V3 sequences in *Oxalis corniculata*. **Table S3.** Identified 16S rDNA sequences of endophytic bacterial isolates in *Alopecurus aequalis*. **Table S4.** Identified 16S rDNA sequences of endophytic bacterial isolates in *Oxalis corniculata*. **Table S5.** Tolerance to each PAH of different endophytic bacteria isolated from *Alopecurus aequalis* roots. **Table S6.** Tolerance to each PAH of different endophytic bacteria isolated from *Alopecurus aequalis* shoots. **Table S7.** Tolerance to each PAH of different endophytic bacteria isolated from *Oxalis corniculata* roots. **Table S8.** Tolerance to each PAH of different endophytic bacteria isolated from *Oxalis corniculata* shoots.
(DOC)
