# Supplementary material for: Distribution of Endophytic Bacteria in Alopecurus aequalis Sobol and Oxalis corniculata L. from Soils Contaminated by Polycyclic Aromatic Hydrocarbons
Source: PLoS One. 2013 Dec 17;8(12):e83054. doi: 10.1371/journal.pone.0083054 (PMC3866203; doi:10.1371/journal.pone.0083054)
Supplement: File S1 — Contains Tables S1–S8. Table S1. Identified 16S rDNA V3 sequences in Alopecurus aequalis. Table S2. Identified 16S rDNA V3 sequences in Oxalis corniculata. Table S3. Identified 16S rDNA sequences of endophytic bacterial isolates in Alopecurus aequalis. Table S4. Identified 16S rDNA sequences of endophytic bacterial isolates in Oxalis corniculata. Table S5. Tolerance to each PAH of different endophytic bacteria isolated from Alopecurus aequalis roots. Table S6. Tolerance to each PAH of different endophytic bacteria isolated from Alopecurus aequalis shoots. Table S7. Tolerance to each PAH of different endophytic bacteria isolated from Oxalis corniculata roots. Table S8. Tolerance to each PAH of different endophytic bacteria isolated from Oxalis corniculata shoots. (DOC) [file pone.0083054.s001.doc]

Table S1. Identified 16S rDNA V3 sequences in *Alopecurus aequalis*

| Tissue | Band No. | Accession No. | Closest NCBI match (Accession No.) | Identity (%) |
| --- | --- | --- | --- | --- |
| **Root** | 1R1-1 | KF051455 | Uncultured bacterium (JX874718.1 ) | 100 |
|  | 1R1-2 | KF051456 | *Pseudomonas mosselii*. (HF952677.1) | 100 |
|  | 1R2-1 | KF051457 | Uncultured bacterium (JX183833.1) | 99 |
|  | 1R4-1 | KF051521 | Uncultured bacterium (HQ910312.1) | 97 |
|  | 1R4-2 | KF051458 | Uncultured bacterium (AB717156.1) | 99 |
|  | 1R6-2 | KF051459 | *Acinetobacter* sp. (KC430970.1) | 99 |
|  | 1R7-1 | KF051460 | Uncultured bacterium (KC764136.1) | 100 |
|  | 1R8-1 | KF051461 | *Halomonas* sp. (AB477015.1) | 100 |
|  | 1R8-2 | KF051461 | Uncultured bacterium (JN641605.1) | 100 |
|  | 1R9-2 | KF051463 | Uncultured bacterium ( FJ152904.1) | 99 |
|  | 1R10-1 | KF051464 | Uncultured bacterium (EU134655.1) | 96 |
|  | 1R11-1 | KF051465 | Bacterium enrichment culture DGGE band ( GU270491.1) | 100 |
|  | 1R12-1 | KF051525 | Uncultured *Bacteroidetes bacterium* (HF564294.1 ) | 99 |
|  | 1R14-1 | KF051466 | Uncultured bacterium (JQ624970.1) | 100 |
|  | 1R16-1 | KF051467 | Uncultured *microorganism* (KC841544.1) | 100 |
|  | 1R16-2 | KF051468 | Uncultured *Halomonas* sp. (HM447733.1) | 99 |
|  | 1R17-1 | KF051469 | Uncultured bacterium (AB636927.1) | 100 |
|  | 1R19-1 | KF051470 | Uncultured *Clostridia bacterium* ( EF434224.1) | 100 |
|  | 1R19-2 | KF051471 | *Halomonas* sp. (DQ644495.1) | 99 |
|  | 1R21-1 | KF051472 | Uncultured bacterium (FN567241.1) | 99 |
|  | 1R22-1 | KF051473 | Uncultured bacterium(JF829071.1) | 100 |
|  | 1R22-2 | KF051474 | *Asaia bogorensis strain* (KC756841.1) | 100 |
|  | 1R23-1 | KF051487 | Uncultured bacterium (HE860554.1) | 99 |
|  | 1R23-2 | KF051474 | Uncultured bacterium (HM312378.1) | 100 |
|  | 1R24-1 | KF051476 | *Halomonas* sp. (JQ044787.1) | 99 |
|  | 1R29-1 | KF051523 | *Raoultella terrigena* strain (KC790281.1) | 100 |
|  | 1R29-2 | KF051477 | Uncultured bacterium (FM873355.1 ) | 100 |
|  | 1R30-1 | KF051478 | Uncultured *eukaryote* clone (HM329207.1) | 99 |
|  | 1R30-2 | KF051479 | *Rhodococcus* sp. (DQ406729.1) | 99 |
|  | 1R31-2 | KF051480 | Uncultured *Ralstonia* sp. (GQ129973.1) | 99 |
|  | 1R32-1 | KF051481 | *Pseudomonas viridiflava* (AY574912.1) | 99 |
|  | 1R33-1 | KF051482 | *Streptomyces aomiensis* (JQ899252.1) | 99 |
|  | 1R34-1 | KF051483 | Uncultured bacterium ( KC797663.1) | 100 |
|  | 1R34-2 | KF051484 | *Bacterium Ellin*5280 (AY234631.1) | 98 |
| **Shoot** | 1S1-1 | KF051485 | *Bacillus fumarioli* (KC354687.1) | 100 |
|  | 1S4-1 | KF051486 | *Pseudomonas* sp. (JQ977574.1 ) | 100 |
|  | 1S4-2 | KF051487 | Uncultured bacterium (HE860554.1) | 99 |
|  | 1S6-1 | KF051531 | Uncultured bacterium (KC758924.1) | 99 |
|  | 1S7-1 | KF051488 | *Microbacterium foliorum* strain (HQ832574.1) | 99 |
|  | 1S8-1 | KF051489 | Uncultured *Ilumatobacter* sp. clone (KC684483.1) | 99 |
|  | 1S8-2 | KF051490 | *Finegoldia magna* (KC311751.1) | 100 |
|  | 1S9-1 | KF051491 | *Pseudomonas extremaustralis* (KC790323.1) | 100 |
|  | 1S11-1 | KF051492 | *Microbacterium* sp. (KC534473.1) | 99 |
|  | 1S12-1 | KF051493 | *Mycobacterium* sp. (JQ396585.1) | 99 |
|  | 1S12-2 | KF051523 | *Raoultella terrigena* (KC790281.1) | 99 |
|  | 1S13-1 | KF051494 | *Brevundimonas olei* (KC534480.1) | 100 |
|  | 1S14-1 | KF051495 | Uncultured bacterium (HQ395893.1) | 96 |
|  | 1S15-1 | KF051496 | *Pseudomonas orientalis* (KC834370.1) | 100 |
|  | 1S15-2 | KF051497 | *Pseudomonas* sp. (KC433656.1) | 100 |
|  | 1S16-1 | KF051498 | *Methylobacterium* sp. (JQ977384.1) | 100 |
|  | 1S16-2 | KF051499 | *Frigoribacterium* sp. (JQ977640.1) | 99 |
|  | 1S17-1 | KF051524 | *Pseudomonas extremaustralis* (KC790323.1) | 100 |
|  | 1S18-1 | KF051500 | Uncultured beta *proteobacterium* (KC602748.1) | 100 |

Table S2. Identified 16S rDNA V3 sequences in *Oxalis corniculata*

| Tissue | Band No. | Accession no. | Closest NCBI match (Accession No.) | Identity (%) |
| --- | --- | --- | --- | --- |
| **Root** | 2R1-1 | KF051502 | Uncultured bacterium (JX183833.1) | 99 |
|  | 2R2-1 | KF051503 | *Pseudomonas orientalis* (KC834370.1) | 99 |
|  | 2R2-2 | KF051504 | *Microbacterium* sp. (JQ977681.1) | 100 |
|  | 2R2-3 | KF051505 | *Pseudomonas* sp. (JN886728.1) | 99 |
|  | 2R3-1 | KF051506 | *Enterobacter* sp. (JQ912527.1) | 100 |
|  | 2R3-2 | KF051507 | *Arthrobacter* sp. (JQ977395.1) | 100 |
|  | 2R4-1 | KF051523 | *Raoultella terrigena* (KC790281.1) | 99 |
|  | 2R7-1 | KF051524 | *Pseudomonas extremaustralis* (KC790323.1) | 99 |
|  | 2R8-1 | KF051508 | *Pseudomonas* sp. (JQ977686.1) | 99 |
|  | 2R9-1 | KF051509 | *Sphingomonas* sp. (KC810834.1 ) | 100 |
|  | 2R9-2 | KF051510 | Uncultured *Actinobacteridae* bacterium (JQ291030.1 ) | 95 |
|  | 2R12-1 | KF051511 | *Nocardioides lianchengensis* (JX841006.1) | 100 |
|  | 2R12-2 | KF051512 | *Plantibacter flavus* (KC790247.1 ) | 99 |
|  | 2R13-1 | KF051524 | *Pseudomonas extremaustralis* (KC790323.1) | 100 |
|  | 2R15-1 | KF051513 | *Pseudomonas putida*(HF952667.1) | 100 |
|  | 2R17-1 | KF051531 | Uncultured bacterium ( KC758924.1 ) | 100 |
|  | 2R19-1 | KF051514 | Uncultured *Pseudoclavibacter* sp. clone (JQ976752.1) | 99 |
|  | 2R19-2 | KF051515 | Uncultured bacterium ( JQ448388.1) | 100 |
|  | 2R20-1 | KF051516 | Uncultured *Burkholderiales* bacterium (EU642367.1) | 97 |
|  | 2R20-2 | KF051517 | Uncultured bacterium (KC484408.1) | 100 |
|  | 2R20-3 | KF051518 | *Pseudomonas* sp. (JQ977216.1) | 100 |
|  | 2R21-1 | KF051519 | Uncultured bacterium (HM050641.1) | 99 |
|  | 2R25-1 | KF051520 | *Nesterenkonia* sp. (KC311650.1 ) | 100 |
|  | 2R25-2 | KF051521 | Uncultured bacterium (HQ910312.1) | 98 |
|  | 2R26-1 | KF051522 | *Halomonas* sp. (KC832321.1) | 100 |
| Shoot | 2S1-1 | KF051523 | *Raoultella terrigena* (KC790281.1) | 100 |
|  | 2S2-1 | KF051524 | *Pseudomonas extremaustralis* (KC790323.1) | 99 |
|  | 2S3-1 | KF051525 | Uncultured *Bacteroidetes* bacterium (HF564294.1) | 99 |
|  | 2S4-1 | KF051502 | *Enterobacter* sp. (JQ912527.1) | 100 |
|  | 2S9-1 | KF051526 | *Microbacterium* sp. ( KC810832.1) | 100 |
|  | 2S10-1 | KF051527 | *Methylobacter* sp. (HF565143.1) | 100 |
|  | 2S12-1 | KF051528 | Uncultured *Sphingomonas* sp. clone (JX568390.1) | 100 |
|  | 2S14-1 | KF051529 | Uncultured bacterium (KC775450.1) | 100 |
|  | 2S14-1 | KF051530 | *Enterobacter* sp. (KC534484.1 ) | 100 |
|  | 2S19-1 | KF051531 | Uncultured bacterium (KC758924.1) | 100 |

Table S3. Identified 16S rDNA sequences of endophytic bacterial isolates in *Alopecurus aequalis*

| Tissue | Isolate No. | Accession No. | Closest NCBI match (Accession No.) | Identity (%) |
| --- | --- | --- | --- | --- |
| **Root** | AF1 | JX994089 | *Bacillus* sp. (GU566326.1) | 99 |
|  | AF2 | JX994090 | *Bacillus* sp. (JN400506.1) | 99 |
|  | AF3 | JX994091 | *Bacillus megaterium* (JX312585.1) | 100 |
|  | AF4 | JX994092 | *Bacillus pseudomycoides* (AB738792.1) | 100 |
|  | AF5 | JX994115 | *Bacillus cereus* (JQ900513.1) | 99 |
|  | AF6 | JX994116 | *Pseudomonas* sp. (JF901709.1) | 99 |
|  | AF7 | JX994117 | *Bacillus aryabhattai* (JN084155.1) | 99 |
|  | AF8 | JX994118 | *Bacillus* sp. (JF901703.1) | 99 |
|  | AF9 | JX994119 | *Pseudomonas* sp. (HQ718413.1) | 99 |
|  | AF10 | JX994103 | Uncultured *Pseudomonas* sp. clone  (JQ9 94180.1) | 100 |
|  | AF11 | JX994104 | *Bacillus pumilus* (JX188071.1) | 100 |
|  | AF12 | JX994099 | *Bacillus simplex* (JF496317.1) | 100 |
|  | AF13 | JX994100 | *Bacillus* sp. (AB735984.1) | 99 |
|  | AF14 | JX994101 | Uncultured *Bacillus* sp. clone (JQ90 4734.1) | 99 |
|  | AF15 | JX994122 | *Bacillus* sp. (JQ917989.1) | 99 |
|  | AF16 | JX994123 | *Paenibacillus* sp. ( EU723825.1) | 99 |
|  | AF17 | JX994105 | *Paenibacillus* sp. (FJ944666.6) | 99 |
|  | AF18 | JX994129 | *Bacillus safensis* (HQ696405.1) | 100 |
|  | AF19 | JX994096 | *Bacillus thuringiensis* (AB738791.1) | 99 |
|  | AF20 | JX994095 | *Bacillus* sp. (JQ956511.1) | 100 |
|  | AF21 | JX994112 | *Bacillus safensis* (JN934391.1) | 100 |
|  | AF22 | JX994113 | *Caulobacter* sp. (JQ659583.1) | 99 |
|  | AF23 | JX994114 | *Bacillus* sp. (JX155396.1) | 100 |
|  | AF24 | JX994126 | *Lysinibacillus fusiformis* (JQ900517.1) | 99 |
|  | AF25 | JX994125 | *Bacillus thuringiensis* (JX280922.1) | 100 |
|  | AF26 | JX994124 | *Bacillus pumilus* (AB741462.1) | 99 |
|  | AF27 | JX994093 | *Pseudomonas viridiflava* (AY574912.1) | 99 |
| **Shoot** | AF8 | JX994118 | *Bacillus* sp. (JF901703.1) | 99 |
|  | AF12 | JX994099 | *Bacillus simplex* (JF496317.1) | 100 |
|  | AF13 | JX994100 | *Bacillus* sp. (AB735984.1) | 99 |
|  | AF14 | JX994101 | Uncultured *Bacillus* sp. clone (JQ90473 4.1) | 99 |
|  | AF19 | JX994096 | *Bacillus thuringiensis* (AB738791.1) | 99 |
|  | AF20 | JX994095 | *Bacillus* sp. (JQ956511.1) | 100 |
|  | AF21 | JX994112 | *Bacillus safensis* (JN934391.1) | 100 |
|  | AF28 | JX994094 | *Bacillus* sp. (AB696843.1) | 99 |
|  | AF29 | JX994097 | *Bacillus thuringiensis* (JF460746.1) | 99 |
|  | AF30 | JX994098 | *Bacillus* sp. (HM771670.1) | 99 |
|  | AF31 | JX994102 | *Bacillus* sp. (DQ448792.1) | 99 |
|  | AF32 | JX994120 | *Pseudomonas koreensis* (JQ579642.1) | 97 |
|  | AF33 | JX994106 | *Bacillus safensis* (JX094950.1) | 100 |
|  | AF34 | JX994107 | *Bacillus cereus* (JX317637.1) | 100 |
|  | AF35 | JX994108 | *Pseudomonas fluorescens* (JN020937.1) | 100 |
|  | AF36 | JX994109 | *Staphylococcus pasteuri* (JX077107.1) | 99 |
|  | AF37 | JX994110 | *Bacillus* sp. ( EU910583.1) | 99 |
|  | AF38 | JX994111 | *Bacillus* sp. (HM352320.1) | 100 |
|  | AF39 | JX994127 | *Rhizobium sp.* (EU184088.1) | *99* |
|  | AF40 | JX994121 | *Pseudomonas viridiflava* (JN084135.1) | 98 |

Table S4. Identified 16S rDNA sequences of endophytic bacterial isolates in *Oxalis corniculata*

| Tissue | Isolate No. | Accession no. | Closest NCBI match (Accession No.) | Identity (%) |
| --- | --- | --- | --- | --- |
| **Root** | CO1 | JX994128 | *Bacillus aryabhattai* (JF951729.1) | 99 |
|  | CO2 | JX994129 | *Bacillus safensis* (HQ696405.1) | 100 |
|  | CO3 | JX994130 | *Bacillus megaterium* (JX312585.1) | 100 |
|  | CO4 | JX994136 | *Bacillus* sp. (HQ222345.1) | 100 |
|  | CO5 | JX994150 | *Bacillus* sp. (GU434676.1) | 100 |
|  | CO6 | JX994155 | *Bacillus* sp. (JN210907.1) | 98 |
|  | CO7 | JX994156 | *Pseudomonas* sp. (HQ222612.1) | 99 |
|  | CO8 | JX994131 | *Bacillus aryabhattai* (JX312579.1) | 100 |
|  | CO9 | JX994132 | *Pseudomonas poae* (HQ406827.1) | 99 |
|  | CO10 | JX994146 | *Bacillus thuringiensis* (AB738791.1) | 100 |
|  | CO11 | JX994149 | Uncultured *Rahnella* sp. clone (GQ179 705.1) | 99 |
|  | CO12 | JX994144 | *Bacillus cereus* (JX293338.1) | 100 |
|  | CO13 | JX994147 | *Bacillus anthracis* (JN700109.1) | 100 |
|  | CO14 | JX994148 | *Bacillus cereus* (JQ900513.1) | 100 |
|  | CO15 | JX994154 | *Pseudomonas* sp. ( JX484804.1) | 98 |
| **Shoot** | CO1 | JX994128 | *Bacillus aryabhattai* (JF951729.1) | 99 |
|  | CO2 | JX994129 | *Bacillus safensis* (HQ696405.1) | 100 |
|  | CO4 | JX994136 | *Bacillus* sp. (HQ222345.1) | 100 |
|  | CO16 | JX994137 | *Bacillus* sp. (JN998403.1) | 99 |
|  | CO17 | JX994138 | *Pseudomonas syringae* (AB680549.1) | 99 |
|  | CO18 | JX994139 | *Pseudomonas migulae* (EU111725.2) | 99 |
|  | CO19 | JX994140 | *Aeromonadaceae* sp. (FJ416492. 1) | 99 |
|  | CO20 | JX994141 | *Pseudomonas* sp. (JX233518.1) | 99 |
|  | CO21 | JX994142 | *Bacillus cereus* (JX006608.1) | 100 |
|  | CO22 | JX994134 | *Pseudomonas fluorescens* (AB680178.1) | 99 |
|  | CO23 | JX994135 | *Sphingomonas* sp. (JF716063.1) | 100 |
|  | CO24 | JX994157 | *Pantoea* sp. (EU816766.1) | 99 |
|  | CO25 | JX994143 | *Rahnella* sp. (JQ864391.1) | 99 |
|  | CO26 | JX994145 | *Stenotrophomonas* sp. (JQ717287.1) | 99 |
|  | CO27 | JX994151 | Uncultured *Achromobacter* sp. clone (GU563751.1) | 99 |
|  | CO28 | JX994152 | *Pseudomonas rhodesiae* (FJ462694.1) | 99 |
|  | CO29 | JX994153 | *Flavobacterium* sp. (DQ778318.1) | 99 |

Table S5. Tolerance to each PAH of different endophytic bacteria isolated from *Alopecurus aequalis* roots

| PAHs | NAP | | ANE | | FLU | | PHE | | PYR | | ANT | | FLA | | BaP | |
| --- | --- | --- | --- | --- | --- | --- | --- | --- | --- | --- | --- | --- | --- | --- | --- | --- |
| Medium | M | L | M | L | M | L | M | L | M | L | M | L | M | L | M | L |
| Isolats﹨C_PAH(_mg·L^-1^) | 100 | 100 | 30 | 30 | 30 | 30 | 30 | 30 | 30 | 30 | 30 | 30 | 30 | 30 | 10 | 10 |
| AF1 | **-** | **+** | **-** | **-** | **-** | **+** | **-** | **+** | **-** | **-** | **-** | **+** | **-** | **-** | **-** | **-** |
| AF2 | **-** | **-** | **-** | **-** | **-** | **+** | **-** | **+** | **-** | **+** | **-** | **+** | **-** | **-** | **-** | **-** |
| AF3 | **-** | **+** | **-** | **-** | **-** | **+** | **-** | **+** | **-** | **+** | **-** | **-** | **-** | **-** | **-** | **+** |
| AF4 | **-** | **-** | **-** | **+** | **-** | **-** | **-** | **-** | **-** | **-** | **+** | **-** | **-** | **+** | **-** | **+** |
| AF5 | **+** | **+** | **+** | **+** | **-** | **+** | **-** | **+** | **+** | **+** | **+** | **+** | **-** | **+** | **-** | **+** |
| AF6 | **-** | **-** | **-** | **+** | **-** | **-** | **+** | **+** | **-** | **+** | **+** | **-** | **+** | **-** | **-** | **+** |
| AF7 | **-** | **-** | **-** | **+** | **-** | **-** | **-** | **-** | **-** | **+** | **-** | **-** | **-** | **+** | **-** | **-** |
| AF8 | **-** | **-** | **-** | **-** | **-** | **+** | **-** | **+** | **-** | **+** | **-** | **+** | **-** | **+** | **-** | **+** |
| AF9 | **-** | **+** | **-** | **+** | **+** | **+** | **-** | **+** | **-** | **-** | **-** | **+** | **-** | **-** | **-** | **+** |
| AF10 | **+** | **+** | **+** | **+** | **+** | **+** | **+** | **+** | **-** | **+** | **+** | **+** | **-** | **+** | **+** | **-** |
| AF11 | **-** | **+** | **-** | **+** | **-** | **+** | **-** | **+** | **+** | **+** | **-** | **+** | **-** | **+** | **-** | **+** |
| AF12 | **-** | **+** | **+** | **+** | **-** | **+** | **-** | **+** | **-** | **-** | **-** | **+** | **-** | **+** | **-** | **+** |
| AF13 | **+** | **+** | **+** | **+** | **-** | **-** | **+** | **+** | **+** | **-** | **+** | **+** | **+** | **+** | **+** | **-** |
| AF14 | **+** | **+** | **+** | **-** | **+** | **+** | **+** | **+** | **+** | **+** | **+** | **-** | **-** | **+** | **-** | **+** |
| AF15 | **-** | **+** | **-** | **+** | **-** | **+** | **-** | **+** | **-** | **+** | **-** | **+** | **-** | **+** | **-** | **-** |
| AF16 | **-** | **+** | **-** | **+** | **-** | **+** | **-** | **+** | **+** | **+** | **-** | **+** | **-** | **-** | **-** | **+** |
| AF17 | **-** | **+** | **-** | **+** | **-** | **+** | **-** | **+** | **-** | **-** | **-** | **-** | **-** | **-** | **-** | **-** |
| AF18 | **-** | **+** | **-** | **+** | **-** | **+** | **+** | **+** | **+** | **-** | **-** | **-** | **-** | **+** | **-** | **-** |
| AF19 | **-** | **+** | **+** | **+** | **+** | **+** | **+** | **+** | **-** | **+** | **-** | **+** | **-** | **+** | **-** | **-** |
| AF20 | **+** | **+** | **+** | **+** | **+** | **-** | **+** | **+** | **+** | **+** | **+** | **+** | **+** | **+** | **-** | **-** |
| AF21 | **+** | **+** | **-** | **+** | **-** | **+** | **-** | **+** | **+** | **-** | **-** | **+** | **-** | **+** | **-** | **+** |
| AF22 | **-** | **+** | **+** | **+** | **-** | **+** | **-** | **+** | **-** | **+** | **-** | **+** | **-** | **+** | **-** | **-** |
| AF23 | **-** | **+** | **-** | **+** | **-** | **+** | **-** | **+** | **-** | **-** | **-** | **+** | **-** | **+** | **-** | **-** |
| AF24 | **+** | **+** | **+** | **+** | **+** | **+** | **-** | **+** | **+** | **-** | **+** | **+** | **+** | **-** | **-** | **-** |
| AF25 | **+** | **+** | **+** | **+** | **-** | **-** | **-** | **+** | **+** | **+** | **+** | **+** | **+** | **-** | **+** | **+** |
| AF26 | **+** | **+** | **+** | **+** | **+** | **+** | **+** | **+** | **-** | **+** | **+** | **+** | **-** | **+** | **-** | **-** |
| AF27 | **-** | **+** | **-** | **-** | **-** | **+** | **-** | **+** | **-** | **-** | **-** | **-** | **-** | **-** | **-** | **-** |

M=MSM medium. L=LB medium

Table S6 Tolerance to each PAH of different endophytic bacteria isolated from *Alopecurus aequalis* shoots

| PAHs | NAP | | ANE | | FLU | | PHE | | PYR | | ANT | | FLA | | BaP | |
| --- | --- | --- | --- | --- | --- | --- | --- | --- | --- | --- | --- | --- | --- | --- | --- | --- |
| Medium | M | L | M | L | M | L | M | L | M | L | M | L | M | L | M | L |
| Isolats﹨C_PAH(_mg·L^-1^) | 100 | 100 | 30 | 30 | 30 | 30 | 30 | 30 | 30 | 30 | 30 | 30 | 30 | 30 | 10 | 10 |
| AF8 | **-** | **-** | **-** | **-** | **-** | **+** | **-** | **+** | **-** | **+** | **-** | **+** | **-** | **+** | **-** | **+** |
| AF12 | **+** | **+** | **-** | **+** | **+** | **+** | **-** | **+** | **-** | **+** | **+** | **+** | **-** | **-** | **-** | **+** |
| AF13 | **+** | **+** | **+** | **+** | **-** | **-** | **+** | **+** | **+** | **-** | **+** | **+** | **+** | **+** | **+** | **-** |
| AF14 | **+** | **+** | **+** | **-** | **+** | **+** | **+** | **+** | **+** | **+** | **+** | **-** | **-** | **+** | **-** | **+** |
| AF19 | **-** | **+** | **+** | **+** | **+** | **+** | **+** | **+** | **-** | **+** | **-** | **+** | **-** | **+** | **-** | **-** |
| AF20 | **+** | **+** | **+** | **+** | **+** | **-** | **+** | **+** | **+** | **+** | **+** | **+** | **+** | **+** | **-** | **-** |
| AF21 | **+** | **+** | **-** | **+** | **-** | **+** | **-** | **+** | **+** | **-** | **-** | **+** | **-** | **+** | **-** | **+** |
| AF28 | **-** | **-** | **-** | **-** | **-** | **+** | **-** | **+** | **-** | **+** | **-** | **+** | **-** | **+** | **-** | **+** |
| AF29 | **+** | **+** | **+** | **+** | **+** | **-** | **+** | **+** | **+** | **+** | **+** | **+** | **+** | **+** | **-** | **-** |
| AF30 | **-** | **+** | **+** | **+** | **+** | **+** | **+** | **+** | **+** | **+** | **+** | **+** | **+** | **-** | **-** | **-** |
| AF31 | **+** | **+** | **+** | **-** | **+** | **+** | **+** | **+** | **+** | **+** | **+** | **-** | **-** | **+** | **-** | **+** |
| AF32 | **-** | **+** | **-** | **+** | **-** | **-** | **-** | **+** | **-** | **-** | **-** | **-** | **-** | **-** | **-** | **-** |
| AF33 | **-** | **-** | **-** | **-** | **+** | **+** | **+** | **+** | **-** | **-** | **-** | **-** | **-** | **-** | **-** | **-** |
| AF34 | **+** | **+** | **+** | **+** | **+** | **+** | **-** | **+** | **-** | **-** | **+** | **-** | **-** | **+** | **-** | **-** |
| AF35 | **-** | **+** | **+** | **+** | **-** | **+** | **-** | **+** | **-** | **-** | **-** | **+** | **-** | **+** | **-** | **+** |
| AF36 | **-** | **+** | **+** | **+** | **-** | **+** | **-** | **+** | **-** | **+** | **-** | **+** | **+** | **-** | **+** | **+** |
| AF37 | **+** | **-** | **-** | **-** | **+** | **-** | **-** | **+** | **-** | **-** | **+** | **-** | **-** | **-** | **+** | **+** |
| AF38 | **+** | **+** | **-** | **+** | **+** | **+** | **-** | **+** | **-** | **+** | **+** | **+** | **-** | **-** | **-** | **-** |
| AF39 | **+** | **+** | **+** | **+** | **+** | **+** | **+** | **+** | **-** | **+** | **+** | **+** | **-** | **+** | **-** | **-** |
| AF40 | **+** | **+** | **+** | **+** | **-** | **-** | **+** | **+** | **-** | **+** | **+** | **+** | **+** | **+** | **-** | **+** |

M=MSM medium. L=LB medium

Table S7. Tolerance to each PAH of different endophytic bacteria isolated from *Oxalis corniculata* roots

| PAHs | NAP | | ANE | | FLU | | PHE | | PYR | | ANT | | FLA | | BaP | |
| --- | --- | --- | --- | --- | --- | --- | --- | --- | --- | --- | --- | --- | --- | --- | --- | --- |
| Medium | M | L | M | L | M | L | M | L | M | L | M | L | M | L | M | L |
| Isolats﹨C_PAH(_mg·L^-1^) | 100 | 100 | 30 | 30 | 30 | 30 | 30 | 30 | 30 | 30 | 30 | 30 | 30 | 30 | 10 | 10 |
| CO1 | **-** | **+** | **-** | **+** | **-** | **-** | **-** | **-** | **-** | **+** | **-** | **+** | **-** | **+** | **-** | **-** |
| CO2 | **-** | **+** | **-** | **+** | **+** | **+** | **-** | **+** | **-** | **-** | **-** | **-** | **-** | **-** | **-** | **-** |
| CO3 | **+** | **+** | **+** | **+** | **+** | **+** | **+** | **+** | **+** | **-** | **+** | **+** | **+** | **+** | **+** | **-** |
| CO4 | **+** | **+** | **-** | **+** | **+** | **+** | **+** | **+** | **-** | **-** | **+** | **+** | **+** | **-** | **-** | **-** |
| CO5 | **-** | **+** | **-** | **+** | **-** | **+** | **-** | **+** | **-** | **+** | **-** | **+** | **+** | **-** | **-** | **+** |
| CO6 | **-** | **+** | **-** | **-** | **-** | **+** | **-** | **+** | **-** | **-** | **-** | **-** | **-** | **-** | **-** | **-** |
| CO7 | **-** | **+** | **-** | **+** | **-** | **+** | **-** | **+** | **-** | **+** | **-** | **-** | **-** | **+** | **-** | **+** |
| CO8 | **+** | **+** | **-** | **+** | **-** | **+** | **-** | **+** | **-** | **+** | **-** | **-** | **-** | **+** | **-** | **+** |
| CO9 | **-** | **+** | **-** | **+** | **-** | **+** | **-** | **+** | **-** | **+** | **-** | **-** | **-** | **+** | **-** | **-** |
| CO10 | **-** | **-** | **-** | **-** | **-** | **-** | **-** | **-** | **-** | **-** | **-** | **-** | **-** | **-** | **-** | **-** |
| CO11 | **-** | **+** | **-** | **+** | **+** | **+** | **-** | **+** | **-** | **-** | **-** | **-** | **-** | **-** | **-** | **+** |
| CO12 | **+** | **+** | **-** | **-** | **-** | **-** | **-** | **-** | **-** | **-** | **-** | **-** | **-** | **-** | **-** | **-** |
| CO13 | **-** | **+** | **+** | **+** | **+** | **+** | **-** | **+** | **-** | **+** | **+** | **+** | **-** | **+** | **-** | **-** |
| CO14 | **+** | **+** | **-** | **+** | **+** | **+** | **-** | **+** | **-** | **+** | **-** | **-** | **-** | **+** | **-** | **-** |
| CO15 | **-** | **+** | **-** | **+** | **-** | **-** | **-** | **-** | **-** | **+** | **-** | **+** | **-** | **+** | **-** | **-** |

M=MSM medium. L=LB medium

Table S8. Tolerance to each PAH of different endophytic bacteria isolated from *Oxalis corniculata* shoots

| PAHs | NAP | | | ANE | | FLU | | PHE | | PYR | | ANT | | FLA | | BaP | |
| --- | --- | --- | --- | --- | --- | --- | --- | --- | --- | --- | --- | --- | --- | --- | --- | --- | --- |
| Medium | M | | L | M | L | M | L | M | L | M | L | M | L | M | L | M | L |
| Isolats﹨C_PAH(_mg·L^-1^) | 100 | | 100 | 30 | 30 | 30 | 30 | 30 | 30 | 30 | 30 | 30 | 30 | 30 | 30 | 10 | 10 |
| CO1 | **-** | **+** | | **-** | **+** | **-** | **-** | **-** | **-** | **-** | **+** | **-** | **+** | **-** | **+** | **-** | **-** |
| CO2 | **-** | **+** | | **-** | **+** | **+** | **+** | **-** | **+** | **-** | **-** | **-** | **-** | **-** | **-** | **-** | **-** |
| CO4 | **+** | **+** | | **-** | **+** | **+** | **+** | **+** | **+** | **-** | **-** | **+** | **+** | **-** | **+** | **-** | **-** |
| CO16 | **-** | **-** | | **-** | **+** | **-** | **-** | **-** | **+** | **-** | **-** | **-** | **-** | **-** | **-** | **-** | **-** |
| CO17 | **-** | **+** | | **-** | **-** | **-** | **-** | **-** | **-** | **-** | **+** | **-** | **-** | **-** | **-** | **-** | **-** |
| CO18 | **-** | **+** | | **-** | **+** | **+** | **-** | **+** | **+** | **+** | **+** | **-** | **+** | **-** | **-** | **-** | **-** |
| CO19 | **-** | **+** | | **-** | **-** | **-** | **-** | **-** | **-** | **-** | **-** | **+** | **-** | **-** | **-** | **-** | **-** |
| CO20 | **-** | **-** | | **-** | **-** | **-** | **-** | **-** | **+** | **-** | **+** | **-** | **-** | **-** | **+** | **-** | **+** |
| CO21 | **-** | **+** | | **+** | **+** | **+** | **+** | **-** | **+** | **-** | **+** | **-** | **+** | **-** | **+** | **-** | **-** |
| CO22 | **-** | **-** | | **-** | **-** | **-** | **+** | **-** | **-** | **-** | **+** | **-** | **-** | **-** | **-** | **-** | **-** |
| CO23 | **-** | **-** | | **-** | **-** | **-** | **-** | **+** | **-** | **-** | **-** | **-** | **-** | **-** | **+** | **-** | **-** |
| CO24 | **+** | **-** | | **-** | **-** | **-** | **-** | **-** | **+** | **-** | **-** | **+** | **+** | **-** | **-** | **-** | **-** |
| CO25 | **-** | **+** | | **-** | **+** | **-** | **-** | **-** | **+** | **-** | **-** | **-** | **-** | **-** | **-** | **-** | **-** |
| CO26 | **+** | **+** | | **+** | **+** | **+** | **+** | **+** | **+** | **+** | **-** | **+** | **+** | **+** | **+** | **+** | **-** |
| CO27 | **-** | **-** | | **-** | **+** | **-** | **-** | **-** | **+** | **-** | **-** | **-** | **-** | **-** | **-** | **-** | **-** |
| CO28 | **-** | **+** | | **-** | **-** | **-** | **-** | **-** | **-** | **-** | **+** | **-** | **-** | **-** | **-** | **-** | **-** |
| CO29 | **-** | **+** | | **-** | **+** | **+** | **-** | **+** | **+** | **+** | **+** | **-** | **+** | **-** | **-** | **-** | **-** |

M=MSM medium., L=LB medium
